# Eco-Evolutionary Processes Generating Diversity Among Bottlenose Dolphin, *Tursiops truncatus*, Populations off Baja California, Mexico

**DOI:** 10.1007/s11692-018-9445-z

**Published:** 2018-01-29

**Authors:** Iris Segura-García, Liliana Rojo-Arreola, Axayácatl Rocha-Olivares, Gisela Heckel, Juan Pablo Gallo-Reynoso, Rus Hoelzel

**Affiliations:** 1 Department of Biosciences, Durham University, South Road, Durham, DH1 3LE UK; 2 CONACYT-Centro de Investigaciones Biológicas del Noroeste (CIBNOR), Mar Bermejo 195, Col. Playa Palo de Santa Rita, 23096 La Paz, BCS Mexico; 3 Centro de Investigación Científica y Educación Superior de Ensenada (CICESE), 22860 Ensenada, Baja California Mexico; 4 Centro de Investigación en Alimentación y Desarrollo, A.C. Unidad Guaymas, Carretera a Varadero Nacional km 66, Col. Las Playitas, 85480 Guaymas, Sonora Mexico

**Keywords:** Bottlenose dolphin, Ecological affinity, Population structure, Stable isotopes, Gulf of California

## Abstract

For highly mobile species that nevertheless show fine-scale patterns of population genetic structure, the relevant evolutionary mechanisms determining structure remain poorly understood. The bottlenose dolphin (*Tursiops truncatus*) is one such species, exhibiting complex patterns of genetic structure associated with local habitat dependence in various geographic regions. Here we studied bottlenose dolphin populations in the Gulf of California and Pacific Ocean off Baja California where habitat is highly structured to test associations between ecology, habitat dependence and genetic differentiation. We investigated population structure at a fine geographic scale using both stable isotope analysis (to assess feeding ecology) and molecular genetic markers (to assess population structure). Our results show that there are at least two factors affecting population structure for both genetics and feeding ecology (as indicated by stable isotope profiles). On the one hand there is a signal for the differentiation of individuals by ecotype, one foraging more offshore than the other. At the same time, there is differentiation between the Gulf of California and the west coast of Baja California, meaning that for example, nearshore ecotypes were both genetically and isotopically differentiated either side of the peninsula. We discuss these data in the context of similar studies showing fine-scale population structure for delphinid species in coastal waters, and consider possible evolutionary mechanisms.

**Electronic supplementary material:**

The online version of this article (10.1007/s11692-018-9445-z) contains supplementary material, which is available to authorized users.

## Introduction

Despite high dispersal potential, many highly mobile marine species show significant population structuring (e.g. Hoelzel 2009) influenced by extrinsic factors such as climatic and oceanographic variation (e.g. Natoli et al. 2005; Fontaine et al. 2007), and intrinsic factors such as site fidelity to specific feeding and breeding grounds (e.g. Baker et al. 1998; Louis et al. 2014). For some cetacean species, ecological specialists may also show differentiation in sympatry (e.g. Hoelzel et al. 2007; Moura et al. 2015). Intraspecific differences in habitat use, in particular among small cetacean species, have been associated with population differentiation of phenotypic and genetic traits (Hoelzel 2002; Natoli et al. 2005; Moura et al. 2014; Louis et al. 2014). Here we consider the potential role of prey choice and fine-scale geographic structure towards the evolution of differentiation among putative populations of the bottlenose dolphin.

The genus *Tursiops* has been one of the most taxonomically controversial among delphinid cetaceans. It exhibits high levels of phenotypic and genotypic polymorphisms resulting in more than 15 nominal species having been described (see Horton et al. 2017). Currently two species, *T. truncatus*, and *T. aduncus* are widely accepted based on morphological and genetic evidence, and a third (*T. australis*) has been recently proposed (Wang et al. 1999; Charlton-Robb et al. 2011; Owen et al. 2011; Moura et al. 2013). However, molecular genetic evidence also shows reciprocal monophyly for the South African and Asian “*aduncus*” forms (Natoli et al. 2004; Moura et al. 2013) and supports other proposed taxonomic units, such as the named subspecies in the Black Sea, *T. truncatus ponticus* (Natoli et al. 2005; Viaud-Martínez et al. 2008). There is also substantial population-level differentiation, for example in the Mediterranean Sea the pattern of population structure suggests the occurrence of at least two habitat dependent populations (Natoli et al. 2005; Gaspari et al. 2015). Fine-scale population structure has also been reported for New Zealand, the Gulf of Mexico, the Caribbean Sea, the Iberian Peninsula, southeast Australia and in the Northern Bahamas (Tezanos-Pinto et al. 2008; Sellas et al. 2005; Caballero et al. 2011; Fernández et al. 2011 and Parsons et al. 2006; Charlton-Robb et al. 2015).

**Fig. 1:**
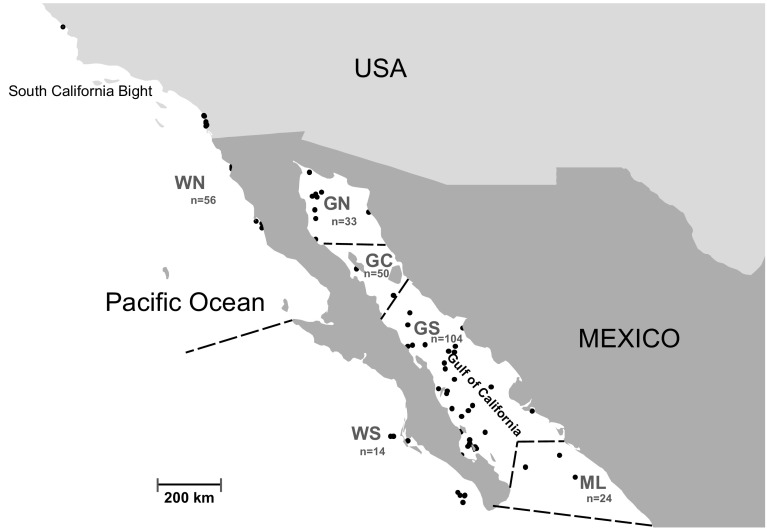
Study area showing all *Tursiops truncatus* samples gathered in this study (N = 281), including new tissue samples and those previously published (Segura et al. 2006). Separate bioregions are delineated by dashed lines. *GN* Gulf of California North, *GC* Gulf of California Central, *GS* Gulf of California South, *ML* Mainland, *WS* West coast Baja California South, *WN* West coast Baja California North

In the Gulf of California (GC) and Southern California bottlenose dolphins have also shown evidence of phenotypic, ecological and genetic differentiation, which supports the recognition of “coastal” and “offshore” ecotypes (Segura et al. 2006; Lowther-Thieleking et al. 2015; Guevara-Aguirre and Gallo-Reynoso 2016), and formerly described as two nominal species (*T. gillii* Dall 1873 and *T. nuuanu* Andrew 1911, respectively; see Segura et al. 2006). Lowther-Thieleking et al. (2015) found significant differentiation among putative nearshore and offshore populations near the Channel Islands at microsatellite loci and mtDNA, Segura et al. (2006) found differentiation between offshore and nearshore populations in the GC based on mtDNA, while Guevara-Aguirre and Gallo-Reynoso (2016) found morphological, habitat use and behavioural differences between nearshore and offshore populations. The GC provides an excellent opportunity to study possible ecological mechanisms shaping population differentiation, as it offers great diversity of habitats (Briggs 1995; Brusca et al. 2005). There are at least four bioregions defined within the gulf reflecting variation in depth, salinity, surface temperature and tidal range (e.g. Santamaría del Angel et al. 1994, Soto-Mardones et al. 1999; Lavín and Marinone 2003), and two defined along the Pacific coast of Baja California either side of Punta Eugenia (e.g. Soto-Mardones et al. 2004, Jacobs et al. 2004; see Fig. 1). Indeed, patterns of genetic structure within the GC, and between GC and the Pacific Ocean off Baja California have been detected in various taxa, including marine invertebrates (Correa-Sandoval and Carvacho 1992; De la Rosa Veléz et al. 2000), fish (Riginos and Nachman 2001; Sandoval-Castillo et al. 2004; Lin et al. 2009), and the California sea lion (Schramm et al. 2009). This reflects the broader biogeographic pattern of the region (Walker 1960; Santamaría-del Ángel et al. 1994; Stepien et al. 2001; Bernardi et al. 2003).

Here we use analyses based on population genetics and stable isotopes to test hypotheses about the process of population differentiation over a relatively small geographic scale (within the gulf and either side of the peninsula) for a highly mobile species in a heterogeneous environment. Specifically, we test the hypothesis that local nearshore and offshore populations either side of the Baja California peninsula will show differentiation for both genetic markers and stable isotopes, indicative of an association between prey choice and population genetic structure. We further test for evidence of structure between ecologically distinct northern and southern portions of the GC and outer Baja California coastal range (see Fig. 1), and the relative importance of demographic factors associated with regional population histories.

## Methods

### Sample Collection and DNA Extraction

A total of N = 281 tissue samples were analysed in this study, comprised of biopsy samples, material from stranded and captive animals and DNA from samples used in an earlier study. Skin biopsy samples (N = 175) were collected from regions within the Gulf of California and along the western coast of Baja California and South California Bight (Fig. 1), using the darting system described by Kellar et al. (2013). Samples collected during 2008–2009 were stored in salt/DMSO for genetic analyses, and a subset in glass or frozen for stable isotope analyses. Tooth samples were obtained from stranded dolphin materials held in the osteological collection of Biology Institute of the National University of Mexico (IBUNAM), but only two of these could be successfully genotyped and sequenced for the full segment (out of 58 teeth, not counted in the total given above). Twenty-one samples were obtained from captive dolphins (Vallarta Adventures and Dolphin Discovery), originally captured off mainland (ML) along the coast of Sinaloa (n = 19), and southwest Baja California (n = 2). Samples collected for the current study were pooled with 83 samples used in a previous study in the GC (Segura et al. 2006). Not all samples amplified successfully for both mtDNA and microsatellite loci (see Table 1).

**Table 1:** *T. tuncatus* sample sizes analyzed for mtDNA control region (dloop), microsatellite loci (msats), and C and N stable isotopes (SIA)

Region	Group	Total	dloop	msats	SIA
Gulf of California North	GNn	32	29	27	–
	GNs	1	1	–	
Gulf of California Central	GCn	11	10	9	9
	GCo	38	36	34	8
	GCs	1	1	–	–
Gulf of California South	GSn	7	3	5	–
	GSo	74	62	62	31
	GSu	23	22	19	–
Mainland	MLn	19	18	17	–
	MLu	5	4	5	–
Baja California West coast South	WSo	3	3	3	1
	WSu	11	11	11	–
Baja California West coast North	WNn	37	35	35	11
	WNu	19	15	19	–
	Total	281	250	246	60

*GN* Gulf of California North-nearshore ecotype, *GCn* Gulf of California Central-nearshore ecotype, *GNs* Gulf of California North-stranding, *GCo* Gulf of California Central-offshore ecotype, *GCs* Gulf of California Central-stranding, *GSn* Gulf of California South-nearshore ecotype, *GSo* Gulf of California South-offshore ecotype, *GSu* Gulf of California South-unknown ecotype, *MLn* Mainland nearshore-ecotype, *MLu* Mainland unknown-ecotype, *WSo* West coast South offshore-ecotype, *WSu* West coast South unknown-ecotype, *WNn* West coast North-nearshore ecotype, *WNu* West coast North-unknown ecotype

When possible, source individuals were categorised as ‘offshore’ or ‘nearshore’ ecotypes using physical characteristics (with the nearshore ecotype being larger and more robust than the offshore, with lighter-coloured dorsal area and flanks, shorter and wider rostrum, relatively shorter and wider flippers and a white belly; after Perrin et al. 2011). Along the coast of California and Baja California offshore dolphins were usually found further than 4 km from shore (Lowther-Thieleking et al. 2015), while nearshore dolphins seem to follow a narrow alongshore corridor less than 1 km wide and in waters less than 60 m depth (Guzón 2002; Morteo et al. 2004). However, most ecotype assignments were done by visual assessment of morphology in the field, rather than by location (see methods described in Segura et al. 2006). Sample sizes by region and ecotype are shown in Table 1. Putative populations were defined by both location and ecotype. DNA was extracted from skin and blood samples following standard protocols (after Aljanabi and Martinez 1997). Tooth DNA extractions were done in ancient DNA facilities taking standard precautions against contamination (see Baker and Hoelzel 2014). Teeth were drilled and DNA extracted from the powder using spin purification columns (QIAGEN, UK).

### Genetic Analyses

Fragments of 480 bp from the mtDNA control region, tRNA proline end, were analysed for 250 samples (see Table 1). The PCRs were performed in 25µL volumes consisting of 10 mM Tris–HCl, 50 mM KCl, 2.5 mM MgCl_2_, 0.25 mM each dNTP, 0.12 µM each primer: L15812 (TRO): 5′ CCT CCC TAA GAC TCA AGG AAG 3′ and H16343 (D): 5′ CCT GAA GTA AGA ACC AGA TG 3′ (Rosel et al. 1994), 1.25 U of Taq DNA polymerase (NEB, UK), and approximately 50 ng of genomic DNA. The cycle profile was 5 min at 95 °C, followed by 36 amplification cycles of 45 s at 48 °C, 1 min at 72 °C and 45 s at 94 °C and a final elongation step of 10 min at 72 °C. PCR products were purified using purification spin columns (QIAGEN, UK) and then sequenced in an automatic sequencer (ABI 3730 Gene Analyzer, Applied Biosystems).

Sequences were checked with the software CHROMAS lite (Technelysiun Pty. Ltd.) to verify base calling and aligned with CLUSTAL X (Jeanmougin et al. 1998). Unique haplotypes were identified using DNAsp version 3 (Rozas and Rozas 1999). The best fit evolutionary model was identified using MODELTEST 3.7 (Posada and Crandall 1998). Haplotype diversity (*h*), nucleotide diversity (*π*), fixation indices (*F*_*st*_ and *ϕ*_*st*_), tests for neutrality (Tajima’s D and Fu’s Fs) and mismatch distributions were estimated using ARLEQUIN 3.5 (Excoffier and Lischer 2010). Estimates of expansion time from tau (after Rogers and Harpending 1992) were based on a generation time estimate of 20 years (Reeves and Notarbartolo di Sciara 2006). The calculation was scaled by mutation rate (µ = 5 × 10^−7^ substitutions/year) for the mtDNA control region HVR1, after Ho et al. (2007). A median-joining network phylogenetic reconstruction of mtDNA haplotypes, rooted with homologous sequences from *Delphinus delphis*, was generated with the program NETWORK 4.5.1.0 (Bandelt et al. 1999).

Eight microsatellite loci: *MK5, AAT44, TexVet5* and *TexVet7*, derived from *T. truncatus* (Rooney et al. 1999; Krützen et al. 2001; Caldwell et al. 2002, respectively), *KWM1b, KMW2b, KWM12a*, derived from *Orcinus orca* (Hoelzel et al. 1998a), and EV37Mn derived from *Megaptera novaeangliae* (Valsecchi et al. 1997), were amplified by PCR. The PCR reactions were performed in 15 µL volumes consisting of 10 mM Tris–HCl, 50 mM KCl, 1.5–2.5 mM MgCl_2_, 0.25 mM each dNTP, 0.12 µM each primer and cycled at 95 °C hot start denaturation followed by 40 cycles of 1 min annealing, 45 s at 72 °C and 45 s at 95 °C, and a final elongation of 10 min at 72 °C. Annealing temperatures were: *MK5*: 53 °C, *AAT44*: 52.6 °C, *TexVet5*: 50 °C, *TexVet7*: 50 °C, *KWM1b*: 49 °C, *KMW2b*: 43 °C, *KWM12a*: 56 °C and *EV37Mn*: 51 °C.

Genotypes across all loci were tested for the presence of allelic dropout and null alleles using the program MICRO-CHECKER (Van Oosterhout et al. 2004). Genotyping strategy was based on replication of 20% of genotypes followed by assessment and when necessary revision of genotype calling strategy. Initial screening revealed a 5% error rate overall, which permitted more accurate subsequent screening. Genetic diversity estimated as observed heterozygosity (*Ho*) and expected heterozygosity (*He*), regional differences in frequencies, deviation from Hardy–Weinberg equilibrium, and F_st_ were all computed in ARLEQUIN 3.5 (Excoffier and Lischer 2010). Allelic richness and the number of alleles per locus were estimated using FSTAT 2.9.3 (Goudet 2002). Population structure was assessed using STRUCTURE 2.3.4 (Pritchard et al. 2000), whereby five independent runs for each putative number of populations (K = 1–9) were performed and checked for consistency, using the correlated allele frequency and admixture models, with 1,000,000 repetitions and a burn-in of 500,000. Population structure was inferred by assessing support for different values of K, including ∆K, which correspond to the highest hierarchical level of structure (Evanno et al. 2005). Structure was also assessed by factorial correspondence analysis (FCA) using the ‘3D by population’ method implemented in GENETIX v. 4.05.2 (Belkhir et al. 2004). Sex was determined by amplifying fragments of the gene *Zfy*/*x* and *SRY* (Pomp et al. 1995). Sex-biased dispersal was tested by estimations of F_IS_, F_ST_, relatedness, mean assignment index and variance of assignment indices using FSTAT 2.9.3 (Goudet 2002).

### Stable Isotope Analyses

A subsample of 60 skin biopsies could be analysed for ^13^C and ^15^N stable isotopes, (see Table 1). Skin samples were dried overnight at 60 °C. Lipid extraction was performed in the extractor Goldfish A-50280 using petroleum ether as organic dissolvent. The tissue was then re-dried, powdered and ~ 1 g of powdered tissue was transferred into tin capsules for mass spectrometry analyses. δ^13^C and δ^15^N were determined using a PDZ Europa ANCA-GSL elemental analyser interfaced to a PDZ Europa 20–20 isotope ratio mass spectrometer at the University of California Davis Stable Isotope Facility. Carbon and nitrogen ratios were expressed in delta notation (δ), in units per mil (McKinney et al. 1950). Delta values are reported relative to the international standards of Vienna Pee-Dee Belemnite carbon and atmospheric nitrogen; the average precision was 0.1 for both δ^13^C and δ^15^N across runs. The C/N mass ratios for each sample are given in table S1. The isotopic niche width for each bottlenose dolphin population was determined based on the isotopic dispersion of samples within a two-dimensional (δ^13^C and δ^15^N) space and significant differences tested using the MANOVA statistic.

## Results

### Genetic Diversity

A total of 34 new mtDNA control region haplotypes were identified among new samples (accession numbers HE617258–HE617297) and pooled with 32 haplotypes from a previous study by Segura et al. (2006) (Genbank accession numbers DQ105702–DQ105733, referred to as TTGC1-32 herein). Collectively 66 mtDNA control region haplotypes were found, defined by 64 segregating sites. No fixed differences were observed across haplotypes from the distinct populations. The best fit evolutionary model was Tamura-Nei with a gamma correction of 0.72, based on AIC and likelihood scores (not shown). Haplotype diversity ranged from 0.772 to 0.956, while nucleotide diversities ranged from 0.004 to 0.019 (Table 2). Only 18 haplotypes were shared among all populations (Table S2).

**Table 2:** Genetic diversity indexes and tests for neutrality and population expansion based on mtDNA control region haplotypes

	GN	GCn	GCo	GSo	ML	WS	WN
Nucleotide div.	0.017 (0.009)	0.019 (0.011)	0.019 (0.009)	0.018 (0.009)	0.011 (0.006)	0.018 (0.01)	0.004 (0.002)
Gene div.	0.885 (0.035)	0.936 (0.051)	0.939 (0.018)	0.959 (0.009)	0.900 (0.045)	0.956 (0.045)	0.772 (0.032)
*tau*	6.102 (1.54–10.38)	6.066 (0.03–91.1)	4.989 (1.30–8.96)	5.416 (1.80–8.57)	0.711 (0.0–1.95)	5.479 (0.0–91.73)	1.445 (0.26–2.32)
Expansion time	15,606	(15,514)	12,759	13,852	1818	(14,013)	3683
D (*p*)	1.33 (0.91)	0.84 (0.83)	− 0.496 (0.37)	− 0.233 (0.48)	− 1.19 (0.11)	− 0.040 (0.52)	0.445 (0.70)
Fs (*p*)	− 0.155 (0.50)	1.32 (0.75)	− 1.95 (0.22)	− 9.644 (0.004)	− 2.45 (0.087)	− 0.62 (0.37)	− 1.04 (0.32)
Raggedness	0.048 (0.73)	0.121 (0.09)	0.031 (0.32)	0.016 (0.51)	0.498 (0.97)	0.049 (0.65)	0.063 (0.37)
Mismatch *p*	0.31	0.054	0.136	0.225	0.968	0.548	0.394

See Table 1 for location abbreviations. Parameters symbols: *π* nucleotide diversity, *h* haplotype diversity, *tau* divergence time, *D* Tajima’s D, *Fs* Fu’s Fs, *p* p-value

The eight microsatellite loci were genotyped for 246 bottlenose dolphin individuals. No allelic dropout was identified by MICRO-CHECKER, however four of eight loci deviated from HWE after Bonferonni correction in at least one population (see Table S3). There was no consistent pattern, but one locus (TexVet7) was out of HWE in three of seven putative populations. However, analyses run with and without TexVet7 showed no difference in pattern or significance (data not shown), and so all eight loci were retained.

### Inferring Population Structure

**Fig. 2:**
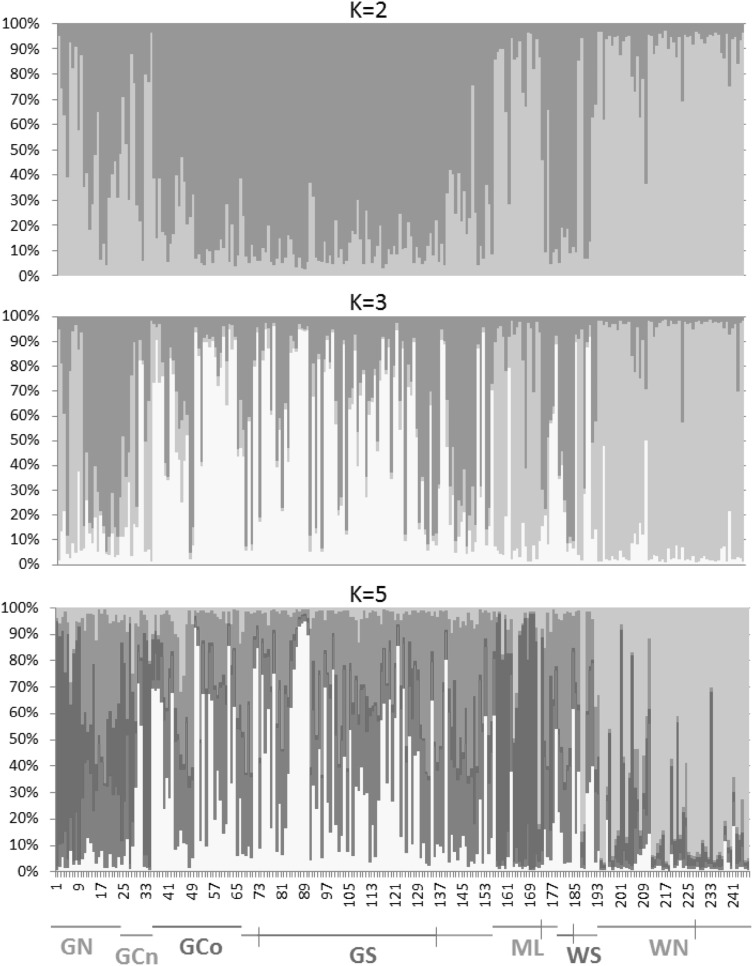
Structure analyses comparing putative *T. truncatus* populations near Baja California. Populations are coded as nearshore (green), offshore (blue) or unknown phenotypes (grey) in the location key below the plots. Plots for K = 2, 3 and 5 are shown. (Color figure online)

**Fig. 3:**
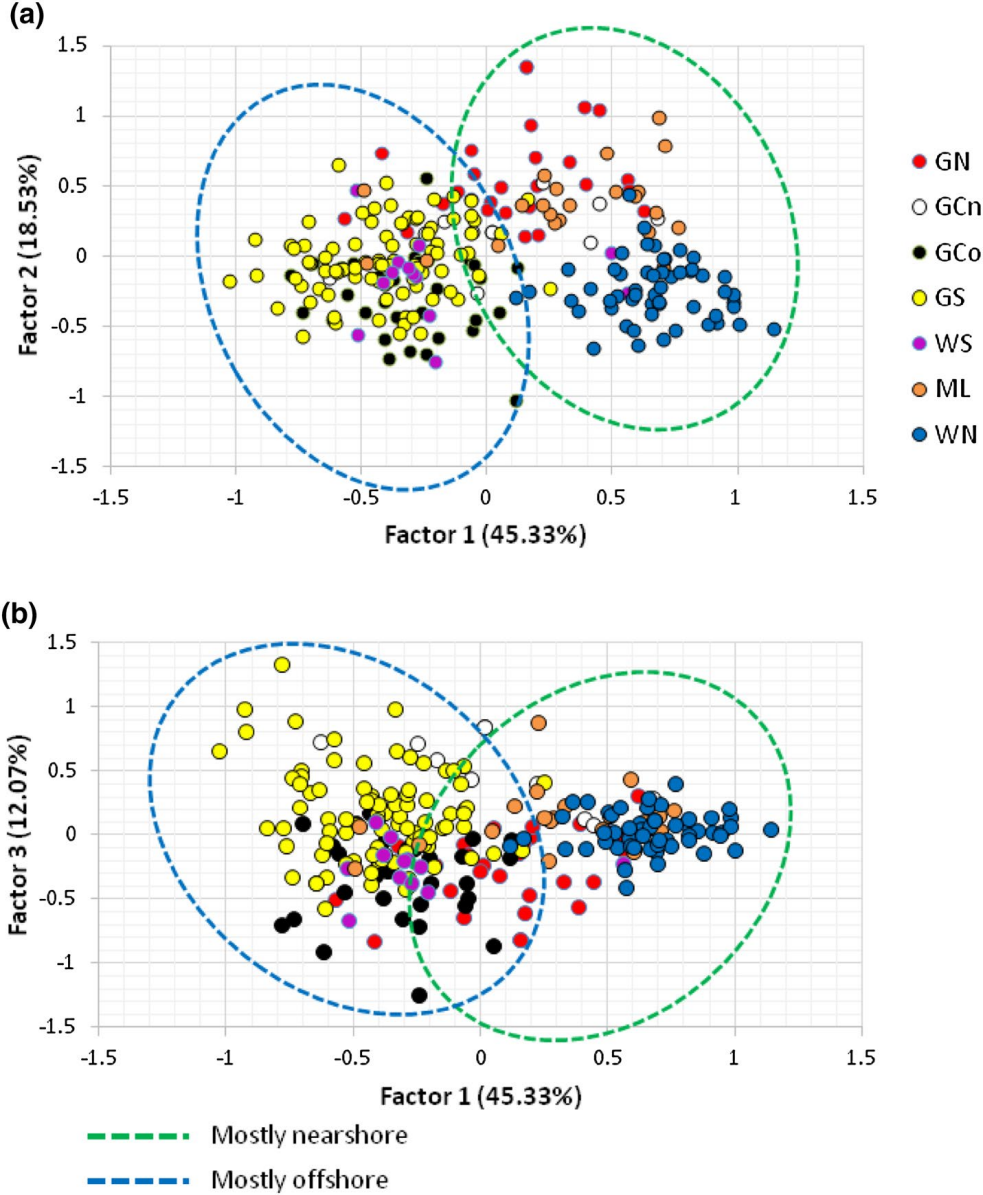
FCA analysis comparing *T. truncatus* samples from the Baja California region (run in Genetix using the 3D by population option). Ellipses illustrate mostly nearshore compared to mostly offshore samples. **a** Comparing factors 1 and 2. **b** Comparing factors 1 and 3. Percent of variance accounted for by each factor is given

The clustering analysis performed in STRUCTURE showed a plateau of similar likelihood values between K = 2 and 5, while ∆K was 2 (see figure S1). Assignment histograms for K = 2, 3 and 5 are shown in Fig. 2. Differentiation both by ecotype and by geography either side of Baja California peninsula is evident. The highest hierarchical level of structure (K = 2 in Fig. 2) largely reflected the distinction between ecotypes. However, the influence of geography can be seen in the differentiation of the northern population on the west side of Baja California (WN) in the STRUCTURE analysis, and more generally in the FCA analyses (Fig. 3). Here again the strongest difference is between the most northern nearshore populations either side of the peninsula, the northern Gulf of California (GN) versus WN. However, the FCA also reveals some level of differentiation between offshore populations from the central (GCo) and southern (GS) Gulf (especially for factor 1 compared to factor 3; Fig. 3b), between nearshore populations in the northern Gulf (GN) and further south along the mainland (ML), and a weaker pattern between GCo and the southern offshore population on the west side (WS; Fig. 3b). There was, however, no evident differentiation between offshore populations either side of the southern end of the peninsula. For those differences only evident in comparisons with factor 3 it should be noted that this factor represented just 12.07% of the variance. Running Structure for each ecotype separately showed structure either side of the peninsula for the nearshores (Figure S2), but K = 1 for the offshores.

**Fig. 4:**
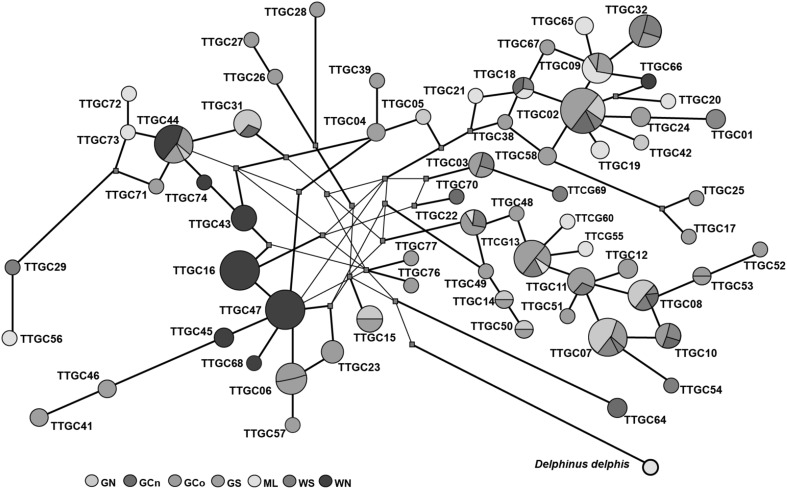
Median neighbor joining network of the 66 *T. truncatus* mtDNA haplotypes sampled within the Gulf of California and western coast of Baja California. The circles represents mtDNA control region haplotypes, the size is proportional to the frequency of the haplotype in the whole dataset

Significant differentiation among populations was seen for both mtDNA (Table 3) and microsatellite DNA data (Table 4), but the pattern indicating the strongest differentiation among ecotypes was most evident for microsatellite DNA data, reinforced by the hierarchical AMOVA analysis (Table 5). The strongest structure was among all populations (F_ST_ = 0.098), followed by differentiation among the two ecotypes (F_CT_ = 0.068) and then differentiation among populations within ecotypes (F_SC_ = 0.032), and all were highly significant (p < 10^−5^, Table 5). For mtDNA the strongest pattern was differentiation between WN compared to the rest, and to some extent ML (Table 3). This was also evident in the structure of the mtDNA network (Fig. 4), and in the distribution of haplotypes (Supplementary Table 1). The statistical tests for sex-biased dispersal showed no significant pattern (data not shown).

**Table 3:** Mitochondrial DNA control region fixation indexes

	*GN*	*GCn*	**GCo**	**GSo**	*ML*	**WS**	*WN*
*GN*		0.076	0.041	0.018	0.274***	− 0.013	0.375***
*GCn*	0.036		0.105***	0.046	0.103	0.095	0.381***
**GCo**	0.023	0.100		0.093***	0.324***	0.045	0.287***
**GSo**	0.026	0.054	0.072		0.144***	0.061	0.304***
*ML*	0.174***	0.152	0.311***	0.144***		0.352***	0.971***
**WS**	− 0.013	0.085	0.035	0.054	0.338***		0.476***
*WN*	0.257***	0.432***	0.283***	0.308***	0.635***	0.455***	

Pairwise comparisons, below diagonal *Fst* and above diagonal *Фst* values, p < 0.008*** after Bonferroni correction. See Table 1 for location abbreviations; italics indicates nearshore ecotype, bold the offshore ecotype

**Table 4:** Microsatellite *Fst* pairwise comparisons, based on eight loci, p < 0.008*** after Bonferroni correction

	*GN*	*GCn*	**GCo**	**GSo**	*ML*	**WS**	*WN*
*GCn*	0.054						
**GCo**	0.046***	0.020					
**GSo**	0.043***	− 0.006	0.010				
*ML*	0.022	0.021	0.039***	0.023***			
**WS**	0.045***	0.041	− 0.010	− 0.008	0.024		
*WN*	0.087***	0.171***	0.107***	0.128***	0.118***	0.124***	

See Table 1 for location abbreviations; italics indicates nearshore ecotype, bold the offshore ecotype

**Table 5:** AMOVA table

Source of variation	Sum of squares	Variance components	% Variation	F-statistics
Among groups	55.471	0.20216	6.77357	F_CT_ = 0.0677
Among populations within groups	37.095	0.08960	3.00211	F_SC_ = 0.0322
Within populations	1204.469	2.69276	90,22433	
Total	1297.034	2.98452		F_ST_ = 0.0978

All F-statistic values significant (p < 0.00001)

### Stable Isotopes

**Fig. 5:**
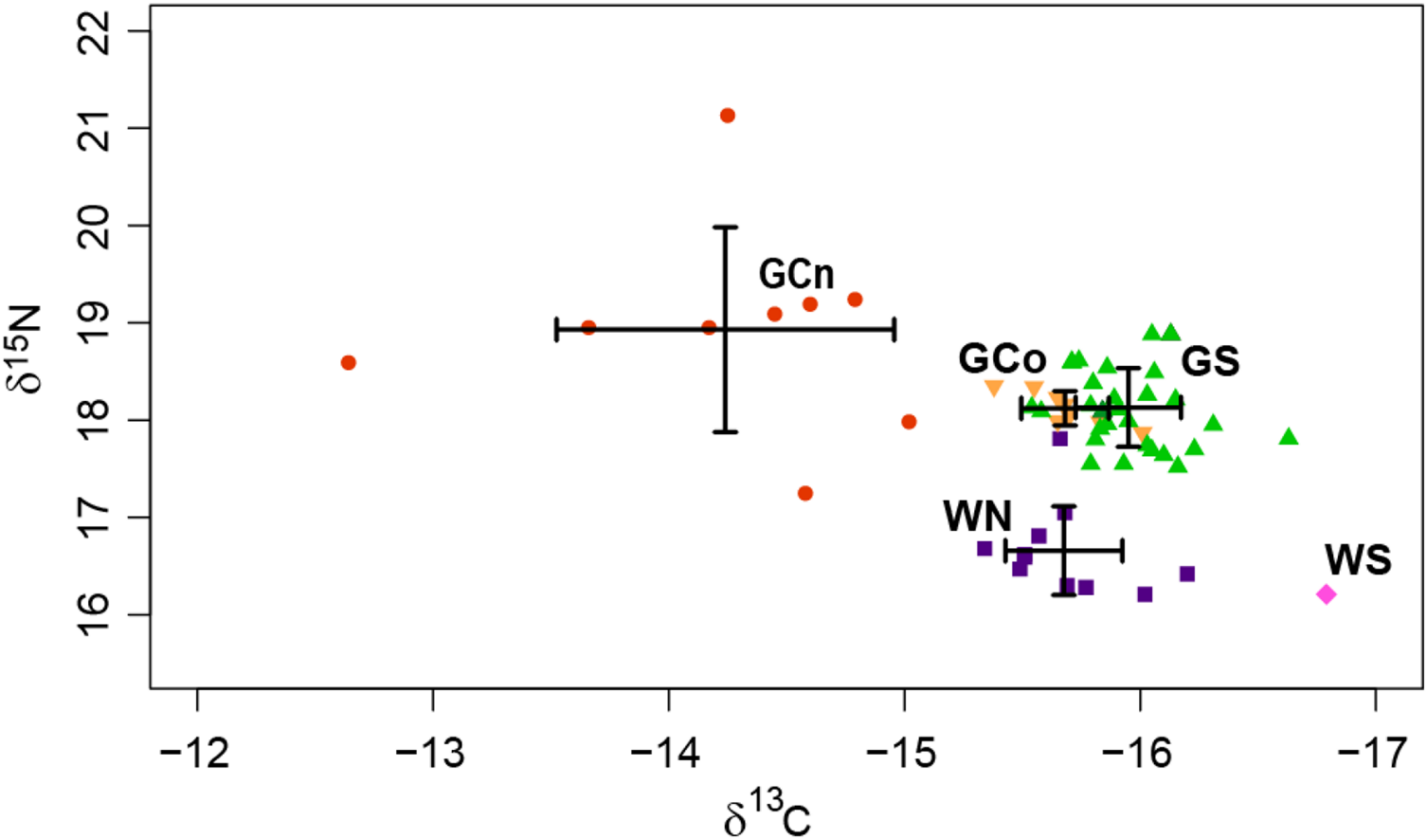
Mean (± SD, ‰) δ13C and δ15N isotope values of four dolphin. *T. truncatus* populations from the Pacific Ocean side: WN (n = 11), WS (n = 1), and within the Gulf of California side: GC-offshore (n = 23), GC-coastal (n = 9). *WN* West coast North, *WS* West coast South, *GC* Gulf of California Central

Although the sample sizes were small and not all putative populations could be sampled, the stable isotope analyses also showed a pattern that reflects differences between nearshore and offshore ecotypes, as well as between populations either side of the peninsula (Fig. 4). Differences among putative populations were significant (MANOVA, Wilk’s lambda = 0.093, F = 29.5, df = 3, p < 0.0001). The samples from the Pacific Ocean coast were relatively depleted for nitrogen, suggesting foraging at lower trophic levels, while the distinction for carbon was inconsistent with either ecotype or geography (Fig. 5).

### Population Dynamics

Signals for population expansion were investigated using tests for neutrality (Tajima 1989; Fu 1997) and mismatch distributions (after Rogers and Harpending 1992) using the mtDNA data. From the neutrality tests, only the GS population suggests evidence for an expansion signal (and only for Fu’s Fs, not Tajima’s D; Table 2). From the mismatch distributions (Figure S3, Table 2) none show significant deviation from the model for expansion, however most appear multimodal (suggesting population stability) except for GS, ML and WN. Two of these populations, ML and especially WN show the lowest diversity (Table 2), consistent with a founder-expansion origin. Estimated expansion times fall into two categories, late Pleistocene/ early Holocene within the gulf, and a more recent event for the nearshore populations WN and ML (Table 2).

## Discussion

### Differentiation Between Populations and Ecotypes

Environmental and ecological factors, such as prey distribution and preference, are increasingly thought to contribute significantly to intra-specific genetic differentiation in mammalian species with high dispersal capabilities (e.g. cetaceans, Hoelzel 1998; felids; McRae et al. 2005; canids, Sacks et al. 2004; Pilot et al. 2006; Muñoz-Fuentes et al. 2009). Differentiation by ecotype in sympatry is well established for various fish species especially based on the use of littoral compared to pelagic or benthic habitats in freshwater lakes for salmonid (see Klemetsen et al. 2003) and various habitats for cichlid species (see Kocher 2004).

Bottlenose dolphin coastal and offshore ecotypes (similar to the aforementioned littoral and pelagic ecotypes) have been previously distinguished within the GC and PO by means of morphological, ecological and mtDNA molecular data (Defran and Weller 1999; Segura et al. 2006; Lowther-Thieleking et al. 2015; Guevara-Aguirre and Gallo-Reynoso 2016). Our data show a pattern of differentiation both between ecotypes (populations inhabiting offshore or nearshore habitat) and over a short geographic range (especially either side of the Baja California peninsula). This was evident for both genetic and stable isotopic markers, suggesting a correspondence between feeding ecology and dispersion. A similar relationship between genetic and stable isotopic data was reported for offshore and nearshore bottlenose dolphin populations in the western North Atlantic (Hoelzel et al. 1998b). Apparent ecological influence on the pattern of differentiation has also been reported for other delphinid species (e.g. spotted dolphin, *Stenella attenuata*, Douglas et al. 1984; Escorza-Treviño et al. 2005; spinner dolphin, *Stenella longirostris*; Perrin and Gilpatrick 1994; Perryman and Westlake 1998; Perrin and Mesnick 2003; tucuxi, *Sotalia fluviatilis*; Caballero et al. 2007; killer whale, *Orcinus orca*; Hoelzel et al. 1998a; and bottlenose dolphin; Bilgmann et al. 2007).

In this study, ^13^C and ^15^N isotopic signals showed evidence of long-term ecological (habitat and prey) affinity within bottlenose dolphin populations. Sampling is unfortunately incomplete, however it is sufficient to show a distinction between profiles for nearshore populations either side of the Baja California peninsula, together with a clear distinction between nearshore and offshore forms within the gulf. There was greater similarity among offshore populations within GC, while the single western offshore sample fell out of that cluster (Fig. 5). The implication is that both geography and prey availability affect their strategies for foraging and prey capture. Different ecotypes show different profiles in sympatry, but the same ecotype differs in allopatry (or parapatry, as in the GC).

The existing biogeographic conditions within the GC are believed to have persisted since the end of the Pleistocene (10,000 years ago; Durham and Allison 1960), thus the Baja California peninsula as a land mass barrier as well as geographical distance per se are the possible factors driving the isolation of bottlenose dolphins from these basins, given that sample localities are separated by hundreds of kilometres. For example, GN and ML populations are separated by approximately 1200 km, straight swimming distance. Several taxa have shown disjunct populations either side of the Baja California peninsula (Stepien et al. 2001; Bernardi et al. 2003; Sandoval-Castillo et al. 2004; Schramm et al. 2009), including species capable of long-distance travelling such as the California sea lion (*Zalophus californianus*). Oceanographic conditions such as dynamic eddies around Punta Eugenia (Soto-Mardones et al. 2004), may impose a boundary to marine species distribution, and this may be particularly important for prey species, indirectly restricting movement of their dolphin predators. Our evidence for stable isotope differentiation between the GS and WS samples would be consistent with this (Fig. 5), though inconclusive due to our having only one WS sample for this comparison.

A study on the feeding ecology of teutophagus cetaceans within the GC revealed that the occurrence of offshore bottlenose dolphins coincided in space and time with that of its preferred prey, the jumbo squid *Dosidicus gigas*, and dolphins were not present in the absence of squid (Díaz-Gamboa 2009). This suggests that movements of offshore bottlenose dolphins across the GC may be coupled to the wide-ranging migratory behaviour of this prey species (Jaquet and Gendron 2002; Rosas-Luis et al. 2008), promoting relatively broad-range connectivity.

At a smaller geographic scale, the clearest distinction was between the nearshore population in the north and offshore populations further south. The sample from the nearshore population in the central gulf (CGn) was small and not clearly differentiated from any of the other gulf populations in our sample set, given the level of resolution obtained. In fact, genetic assignment analyses suggest they may represent a mixture of GN and GCo individuals (see Fig. 2). Possible misidentification in the field was also suggested by a small number of GS individuals, identified as nearshore phenotype, but clustering with offshore types (Table 1; Fig. 2). These factors will have affected our ability to resolve structure at this scale.

The distinctiveness of populations residing in the northern Gulf of California has also been described for several other taxa including fish (Walker 1960; Riginos and Nachman 2001; Lin et al. 2009), crustaceans (Correa-Sandoval and Rodriguez-Cortes 1998), and pinnipeds (Schramm et al. 2009), suggesting a well-defined bioregion possibly delimited by the abrupt change in the temperature and bathymetry at the sills of the Midriff Island (Lopez et al. 2006). Both Sea surface temperature (SST) and depth are oceanographic factors that modify cetaceans travelling routes, as recorded in bottlenose dolphins equipped with radio-transmitters in the Atlantic Ocean (Wells et al. 1999). In the GN, *T. truncatus* preferably inhabits coastal shallow waters of 15–21 °C SST, with high turbidity. *T. truncatus* is also the only cetacean known to venture into the Colorado River; while common dolphins remain in deeper, less turbid waters (Silber et al. 1994). Habitat differences appear to influence the feeding behaviours exhibited by bottlenose dolphins (Torres et al. 2003; Rosel et al. 2009; Torres and Read 2009); for instance a feeding strategy known as intentional beaching has been observed in bottlenose dolphins from the Colorado River (Silber and Fertl 1995). Recent studies have suggested the matrilineal transmission of foraging specializations (Krützen et al. 2005; Sargeant et al. 2005; Weiss 2006) which could promote habitat fidelity and the retention of learned behavioural strategies (e.g. Rosel et al. 2009), thereby restricting gene flow. In addition, in the GN, Silber et al. (1994) reported the occurrence of bottlenose dolphins all year round, with some seasonal movements along the Baja California and Sonora coastline, consistent with a high level of habitat fidelity. Capture-recapture studies using photo-ID have also shown a certain level of residency of coastal dolphins along the eastern and western shores of the GC (e.g. Balance 1990; Reza-García 2001).

The strongest differences are between the northern nearshore population on the western side (WN) and all other putative populations, including the nearshore population within the GC. The WN population is also the strongest candidate for having a founder expansion origin (relatively low diversity and a clear expansion signal from the mismatch distribution, Table 2, Supplementary Fig. 2). Haplotypes identified in our study match those reported in Lowther-Thieleking et al. (2015), indicating some level of continuity between the northern Baja California population (WN) and the southern Californian population off San Diego. Photo-identification studies have also shown that coastal bottlenose dolphins from northern Baja California travel northwards to the Southern California Bight and even as far as Monterey Bay (Hwang et al. 2014). The nearshore mainland population further south along the Mexican coast (ML) is differentiated from nearby populations of both ecotypes (e.g. see Fig. 3). This complex pattern of fine-scale population structure associated with habitats, ecology and natural barriers is consistent with patterns seen for this species elsewhere, such as on both sides of the North Atlantic (e.g. Natoli et al. 2005; Díaz-López and Bernal-Shirai 2008; Louis et al. 2014).

### Population History and Dynamics

For two putative populations with small sample sizes (WS and GCn) the confidence limits on tau were too broad for useful expansion time estimates (Table 2). However, among the rest there were two periods indicated, one between the last glacial maximum and the start of the Holocene for populations within the gulf, and a more recent expansion time (a few 1000 years ago) for the nearshore, WN and ML populations. The earlier dates are consistent with regional oceanic transitions proposed to have played a role in the founding of a common dolphin (*Delphinus capensis*) population in the Gulf of California (see discussion in Segura-García et al. 2016). The later dates span a period that includes the Holocene climatic optima 2 and the Roman warm period, together with an intervening cold period, also proposed to have impacted oceanic current systems (see McMichael 2012). Unlike the case of the common dolphins (Segura-García et al. 2016), differentiation between *T. truncatus* population in and out of the gulf has not generated reciprocally monophyletic lineages through lineage sorting, though there is some mtDNA lineage differentiation between the nearshore population WN and the gulf populations (Fig. 4).

### Conservation and Management Implications

This study revealed a complex pattern of fine-scale population structure for the bottlenose dolphin *Tursiops truncatus* in the GC and the Pacific Ocean off Baja California, and suggests possible mechanisms for the evolution of this structure. The GC has been recognized as a priority for conservation and management actions, given the outstanding levels of biodiversity present in this marginal sea. It is also a region that has seen recent differentiation and incipient speciation for a diversity of taxa (see Segura-García et al. 2016). Our data indicate that the northern GC is of particular concern, as a number of studies consistently indicate the isolation of this region and the need for this to be considered as a critical habitat for a number of species. There are various endemic species at risk of extinction (Lluch-Cota et al. 2007) and the Mexican authorities are currently conducting conservation and management actions in this region. For example, the Vaquita (*Phocoena sinus*) and totoaba (*Totoaba macdonaldi*) are endangered endemic species currently under management actions for population recovery (Barlow et al. 2010). Effective conservation depends on accurate information about stock boundaries, abundance and habitat requirements. It is also important that the relevant evolutionary mechanisms generating structure (such as local adaptation and genetic drift) are understood so that transferable inference can support broader conservation strategies. Our data indicate an influence from both phenotypic adaptation and genetic drift determining fine-scale population structure for a highly mobile marine mammal.

## Electronic supplementary material

Below is the link to the electronic supplementary material.

Supplementary material 1 (DOCX 611 KB)
