# Supplementary material for: Eco-Evolutionary Processes Generating Diversity Among Bottlenose Dolphin, Tursiops truncatus, Populations off Baja California, Mexico
Source: Evol Biol. 2018 Jan 29;45(2):223–36. doi: 10.1007/s11692-018-9445-z (PMC5938318; doi:10.1007/s11692-018-9445-z)

Supplementary Tables & Figures

Table S1. Carbon and nitrogen (C/N) mass ratio estimated from *Tursiops truncatus* skin samples from the Gulf of California and West coast of Baja California.

| ID | Micro g N | Micro g C | C/N |
| --- | --- | --- | --- |
| BGC-13 | 243.8 | 712.5 | 2.923 |
| BGC-34 | 232.7 | 692.3 | 2.975 |
| BGC-22 | 222.7 | 668.4 | 3.002 |
| BGC-71 | 240.5 | 716.2 | 2.978 |
| BGC-75 | 218.0 | 646.3 | 2.965 |
| BGC-18 | 214.5 | 654.5 | 3.051 |
| BGC-10 | 210.0 | 634.3 | 3.020 |
| BGC-17 | 229.8 | 695.1 | 3.024 |
| BCG-09 | 217.7 | 669.3 | 3.074 |
| BGC-12a | 223.6 | 679.4 | 3.039 |
| BGC-24 | 224.7 | 675.7 | 3.008 |
| BGC-27 | 203.7 | 631.5 | 3.101 |
| BGC-33 | 216.6 | 656.4 | 3.031 |
| BGC-38 | 208.5 | 635.2 | 3.047 |
| BGC-74 | 203.7 | 619.6 | 3.042 |
| BGC-77 | 197.7 | 627.9 | 3.175 |
| BGC-78 | 204.7 | 638.0 | 3.116 |
| BGC-81 | 221.4 | 665.6 | 3.006 |
| BGC-12b | 218.2 | 658.2 | 3.017 |
| BGC-84 | 216.0 | 643.5 | 2.978 |
| BGC-21 | 206.9 | 628.8 | 3.039 |
| BGC-79 | 199.9 | 601.2 | 3.007 |
| BGC-07 | 165.4 | 540.4 | 3.267 |
| BGC-85 | 184.3 | 579.1 | 3.143 |
| BGC-86 | 215.0 | 653.6 | 3.041 |
| BGC-19 | 181.6 | 563.4 | 3.103 |
| BGC-02 | 211.2 | 652.7 | 3.090 |
| BGC-16 | 153.0 | 495.3 | 3.237 |
| BGC-11 | 222.5 | 683.1 | 3.070 |
| BGC-14 | 189.6 | 593.8 | 3.131 |
| BGC-15 | 194.0 | 596.6 | 3.076 |
| BGC-76 | 210.1 | 643.5 | 3.063 |
| BGC-23 | 213.9 | 661.9 | 3.095 |
| BGC-03 | 192.9 | 591.0 | 3.064 |
| BGC-08 | 180.0 | 563.4 | 3.131 |
| BGC-83 | 202.6 | 629.7 | 3.108 |
| BGC-20 | 205.3 | 635.2 | 3.094 |
| BGC-36 | 215.0 | 654.5 | 3.045 |
| BGC-37 | 204.2 | 626.0 | 3.066 |
| BCMarzo | 198.3 | 605.8 | 3.055 |
| BBAbr | 218.2 | 656.4 | 3.008 |
| BCMayo | 217.1 | 641.7 | 2.955 |
| BGC-58 | 210.7 | 641.7 | 3.046 |
| G1 | 115.4 | 357.4 | 3.098 |
| G2 | 126.0 | 401.8 | 3.188 |
| G3 | 101.2 | 340.1 | 3.362 |
| BCS01 | 95.9 | 319.1 | 3.326 |
| BTS13 | 88.1 | 298.0 | 3.383 |
| BTS10 | 89.9 | 302.2 | 3.363 |
| SQ1 | 164.0 | 560.3 | 3.417 |
| SQ4 | 96.4 | 344.0 | 3.569 |
| SQ6 | 128.6 | 447.3 | 3.477 |
| SQ11 | 153.0 | 530.4 | 3.467 |
| SQ15 | 89.5 | 319.1 | 3.566 |
| SQ8 | 92.1 | 318.7 | 3.462 |
| SQ10 | 80.3 | 284.5 | 3.544 |
| SQ18 | 92.1 | 322.7 | 3.503 |
| SQ9 | 119.5 | 416.7 | 3.488 |

Table S2. Haplotype frequency by populations. See Table 1 for population abbreviations.

| Haplotype | Accession number | GN | GCn | GCo | GS | WS | ML | WN |
| --- | --- | --- | --- | --- | --- | --- | --- | --- |
| TTCG01 | DQ105702 |  |  |  | 4 |  |  |  |
| TTGC02 | DQ105703 | 4 | 1 |  | 11 | 2 | 4 |  |
| TTGC03 | DQ105704 |  |  | 1 | 3 | 1 |  |  |
| TTGC04 | DQ105705 |  |  |  | 2 |  |  |  |
| TTGC05 | DQ105706 | 1 |  |  |  |  |  |  |
| TTGC06 | DQ105707 |  |  | 4 | 5 |  |  |  |
| TTGC07 | DQ105708 | 8 |  | 6 | 5 | 1 |  |  |
| TTGC08 | DQ105709 | 3 | 2 |  | 1 | 1 |  |  |
| TTGC09 | DQ105710 |  |  | 1 | 3 |  | 6 |  |
| TTGC10 | DQ105711 |  | 1 | 2 | 1 |  |  |  |
| TTGC11 | DQ105712 |  |  |  | 5 | 1 |  |  |
| TTGC12 | DQ105713 |  |  |  | 6 |  |  |  |
| TTGC13 | DQ105714 |  |  | 3 | 6 | 3 | 1 |  |
| TTGC14 | DQ105715 | 1 |  |  | 1 |  |  |  |
| TTGC15 | DQ105716 | 3 |  |  | 3 |  |  |  |
| TTGC16 | DQ105717 |  |  |  |  |  |  | 16 |
| TTGC17 | DQ105718 |  |  |  | 1 |  |  |  |
| TTGC18 | DQ105719 |  | 1 |  | 1 |  | 1 |  |
| TTGC19 | DQ105720 |  |  |  |  |  | 2 |  |
| TTGC20 | DQ105721 |  |  |  |  |  | 1 |  |
| TTGC21 | DQ105722 |  |  |  |  |  | 1 |  |
| TTGC22 | DQ105723 |  |  | 4 | 1 |  | 1 |  |
| TTGC23 | DQ105724 |  |  | 4 |  |  |  |  |
| TTGC24 | DQ105725 |  |  |  | 3 |  |  |  |
| TTGC25 | DQ105726 |  |  |  | 1 |  |  |  |
| TTGC26 | DQ105727 |  |  | 1 |  |  |  |  |
| TTGC27 | DQ105728 |  |  | 1 |  |  |  |  |
| TTGC28 | DQ105729 |  |  | 1 |  |  |  |  |
| TTGC29 | DQ105730 |  |  |  |  | 1 |  |  |
| TTGC30 | DQ105731 | 1 |  |  |  |  |  |  |
| TTGC31 | DQ105732 | 5 | 1 |  |  |  |  |  |
| TTGC32 | DQ105733 |  | 2 | 2 | 5 |  |  |  |
| TTGC38 | HE617258 |  |  |  | 1 |  |  |  |
| TTGC39 | HE617259 |  |  |  | 1 |  |  |  |
| TTGC41 | HE617261 |  |  |  | 1 |  |  |  |
| TTGC42 | HE617262 | 1 |  |  |  |  |  |  |
| TTGC43 | HE617263 |  |  |  |  |  |  | 7 |
| TTGC44 | HE617264 | 1 |  | 3 | 2 |  |  | 6 |
| TTGC45 | HE617265 |  |  |  |  |  |  | 3 |
| TTGC46 | HE617266 |  |  |  | 2 |  |  |  |
| TTGC47 | HE617267 |  |  |  |  |  |  | 16 |
| TTGC48 | HE617268 |  |  |  | 1 |  |  |  |
| TTGC49 | HE617269 |  |  |  | 1 |  |  |  |
| TTGC50 | HE617270 | 1 |  |  | 1 |  |  |  |
| TTGC51 | HE617271 |  |  |  | 1 |  |  |  |
| TTGC52 | HE617272 |  |  |  | 1 |  |  |  |
| TTGC53 | HE617273 |  |  | 1 | 1 |  |  |  |
| TTGC54 | HE617274 |  |  |  |  | 1 |  |  |
| TTGC55 | HE617275 |  |  |  |  |  | 1 |  |
| TTGC56 | HE617276 |  |  |  |  |  | 1 |  |
| TTGC57 | HE617277 |  |  |  | 1 |  |  |  |
| TTGC58 | HE617278 |  |  |  | 2 |  |  |  |
| TTGC59 | HE617279 | 1 |  |  |  |  |  |  |
| TTGC64 | HE617284 |  | 3 |  |  |  |  |  |
| TTGC65 | HE617285 |  |  |  |  |  | 2 |  |
| TTGC66 | HE617286 |  |  |  |  |  | 1 |  |
| TTGC67 | HE617287 |  |  |  | 1 |  |  |  |
| TTGC68 | HE617288 |  |  |  |  |  |  | 1 |
| TTGC69 | HE617289 |  |  |  |  | 1 |  |  |
| TTGC70 | HE617290 |  | 1 |  |  |  |  |  |
| TTGC71 | HE617291 |  |  |  | 1 |  |  |  |
| TTGC72 | HE617292 |  |  |  |  | 1 |  |  |
| TTGC73 | HE617293 |  |  |  |  | 1 |  |  |
| TTGC74 | HE617294 |  |  |  |  |  |  | 1 |
| TTGC76 | HE617296 |  |  | 1 |  |  |  |  |
| TTGC77 | HE61729 |  |  |  | 1 |  |  |  |

Table S3. Genetic diversity at microsatellite loci for each population. n= sample size, Ho: Observed heterozygosity, He: expected heterozygosity, H-W: significance for deviation from H-W (bold significant after Bonferoni correction). E= genotyping error rate. See Table 1 for population abbreviations.

| Populations | | | | | | | | | | |
| --- | --- | --- | --- | --- | --- | --- | --- | --- | --- | --- |
| *Locus* | | **GN**  **n=27** | | **GCn**  **n=9** | **GCo**  **n=34** | **GS**  **n=86** | **ML**  **n=22** | **WS**  **n=14** | | WN  n=54 |
| Tex Vet 5  E= 0.1429 | *Ho*  *He*  H-W | 0.920  0.841  0.427 | 0.286  0.747  0.018 | | 0.793  0.866  0.231 | 0.781  0.886  0.009 | 0.636  0.671  0.242 | 0.667  0.873  0.068 | 0.420  0.448  0.115 | |
| KWM12a  E= 0 | *Ho*  *He*  H-W | 0.808  0.805  0.159 | 0.818  0.796  0.859 | | 0.862  0.859  0.839 | 0.759  0.832  0.029 | 0.714  0.778  0.051 | 0.545  0.749  0.111 | 0.760  0.701  0.927 | |
| KWM2b  E= 0 | *Ho*  *He*  H-W | 0.115  0.148  1 | 0.364  0.311  1 | | 0.469  0.442  1 | 0.515  0.497  0.656 | 0.143  0.219  0.236 | 0.333  0.377  0.399 | 0.440  0.509  0.068 | |
| KWM1b  E= 0.0102 | *Ho*  *He*  H-W | 0.407  0.445  1 | 0.182  0.173  1 | | 0.629  0.526  0.323 | 0.567  0.523  0.766 | 0.000  0.226  **0.003** | 0.384  0.563  0.208 | 0.149  0.209  0.099 | |
| AAT44  E= 0.0204 | *Ho*  *He*  H-W | 0.555  0.586  1 | 0.500  0.516  0.592 | | 0.731  0.756  0.029 | 0.743  0.770  0.006 | 0.500  0.588  0.133 | 0.769  0.786  0.316 | 0.708  0.752  0.083 | |
| MK5  E= 0.0408 | *Ho*  *He*  H-W | 0.833  0.889  0.016 | 0.889  0.830  0.971 | | 0.759  0.859  0.211 | 0.883  0.878  0.355 | 0.700  0.851  0.608 | 0.769  0.834  0.562 | 0.686  0.781  0.165 | |
| Tex Vet 7  E= 0.0816 | *Ho*  *He*  H-W | 0.667  0.655  0.828 | 0.600  0.742  0.023 | | 0.423  0.574  **0.002** | 0.551  0.607  0.522 | 0.250  0.583  **0.002** | 0.461  0.732  0.129 | 0.509  0.565  0.005 | |
| EV37Mn  E= 0.1122 | *Ho*  *He*  H-W | 0.846  0.857  0.108 | 0.889  0.908  0.407 | | 0.806  0.932  0.008 | 0.867  0.945  0.001 | 0.857  0.903  0.215 | 0.727  0.957  0.022 | 0.558  0.659  0.002 | |

Figure S1: Values for delta K (left axis) and Ln(PrK) (right axis) for different values of K (population number) from the analysis in STRUCTURE.

Figure S2: Structure analysis comparing only the nearshore *Tursiops truncatus* around Baja California. A) plot of the LnP(K) and ΔK values for K=1-5. B) Structure plot for K=2. C) Structure plot for K=3. Populations abbreviations are as in Figure 1.

A)

B)

C)

Figure S3: Mismatch distributions for each regional population. See Table 1 for population key.


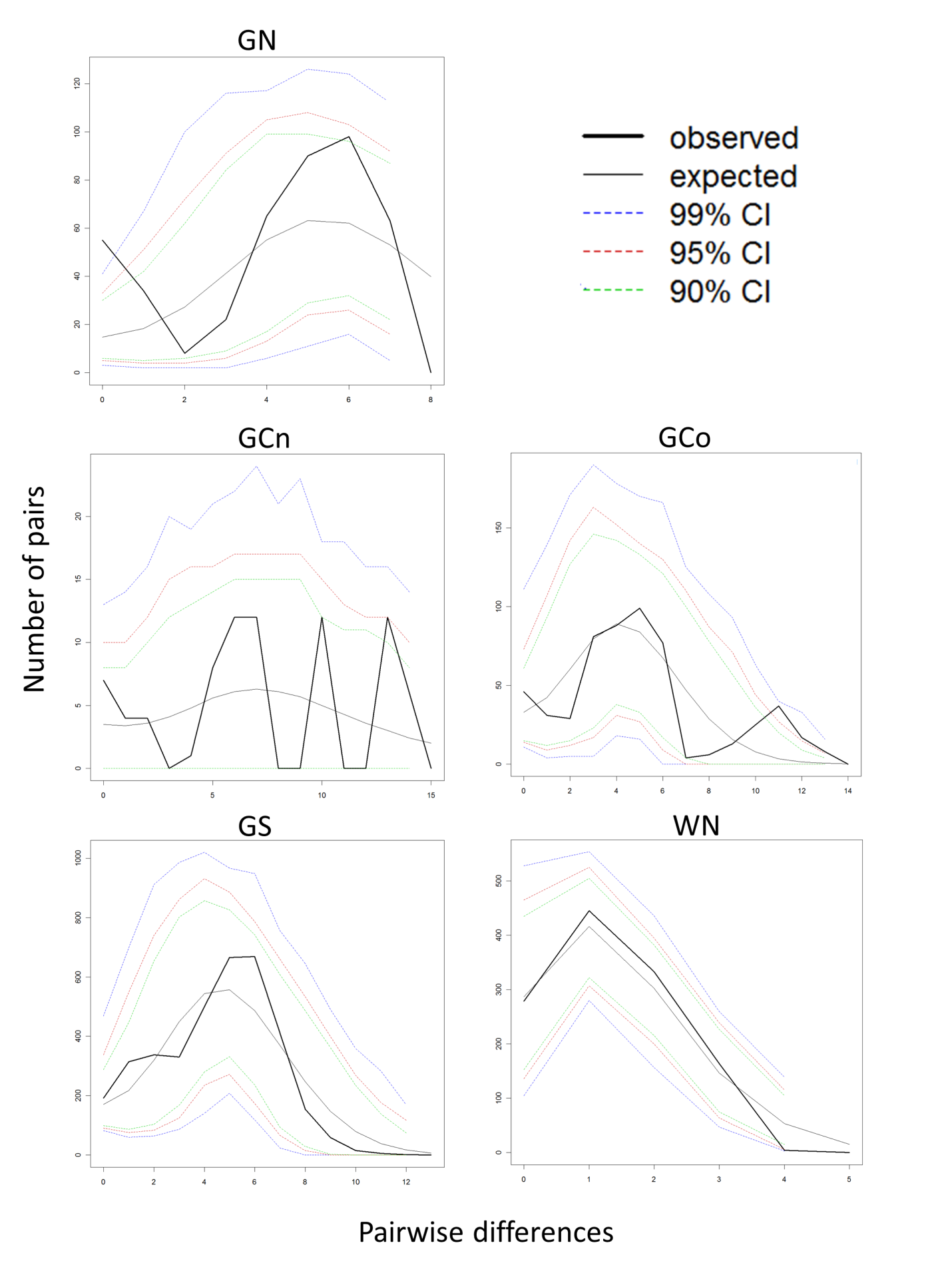


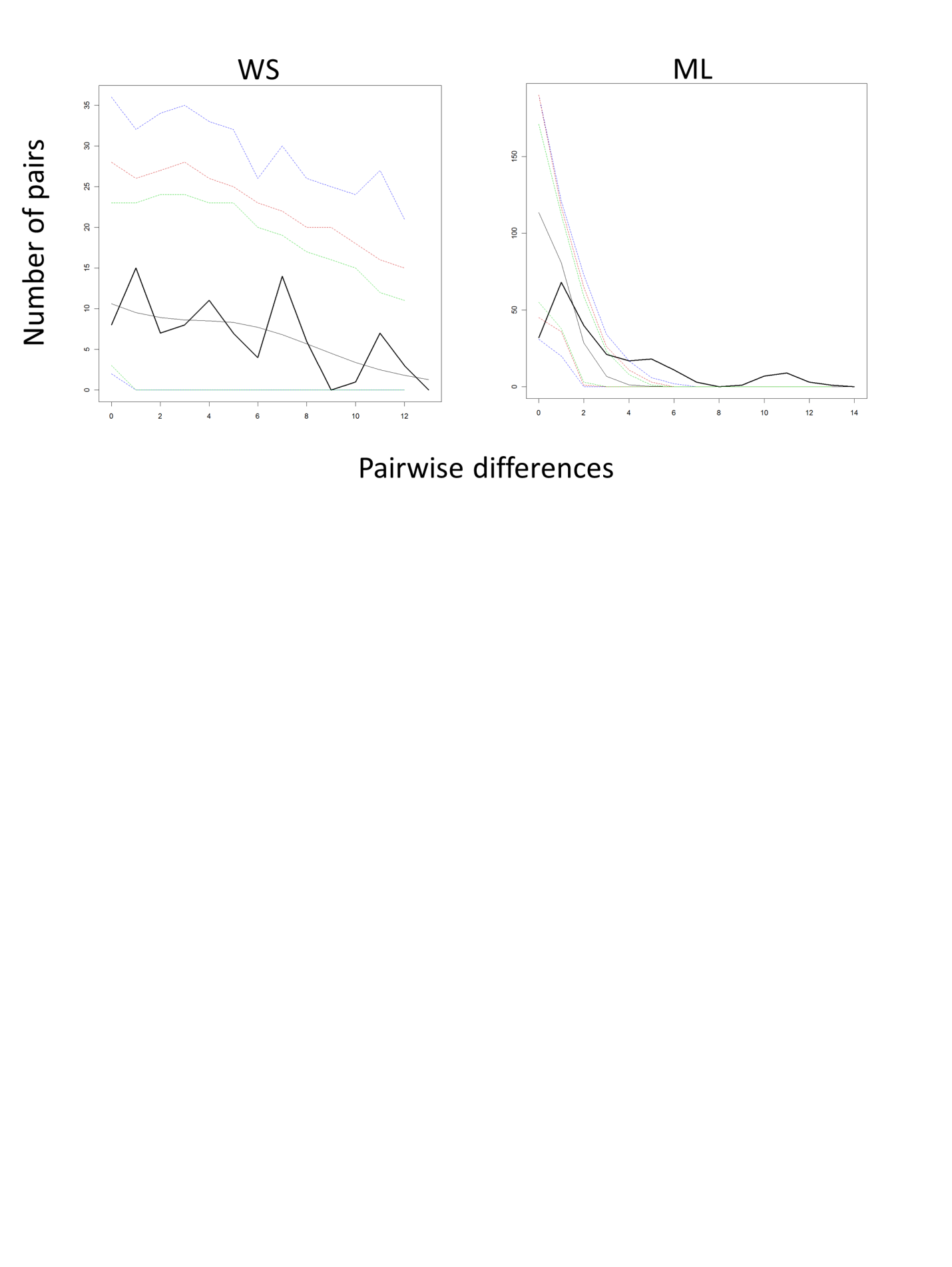

Supplement: Supplementary file 1 — Supplementary material 1 (DOCX 611 KB) [file 11692_2018_9445_MOESM1_ESM.docx]
